# Supplementary material for: Isolation may select for earlier and higher peak viral load but shorter duration in SARS-CoV-2 evolution
Source: Nat Commun. 2023 Nov 21;14:7395. doi: 10.1038/s41467-023-43043-2 (PMC10663562; doi:10.1038/s41467-023-43043-2)
Supplement: Supplementary file 1 — Supplementary Information [file 41467_2023_43043_MOESM1_ESM.pdf]

Supplementary Information

Isolation may select for earlier and higher peak viral load but shorter duration in SARS-CoV-2 evolution

Junya Sunagawa[1,†], Hyeongki Park[2,†], Kwang Su Kim[2,3,4,†], Ryo Komorizono[5], Sooyoun Choi[2,4], Lucia Ramirez Torres[2], Joohyeon Woo[2], Yong Dam Jeong[2,4], William S Hart[6], Robin N. Thompson[6,7,8], Kazuyuki Aihara[9], Shingo Iwami[2,10,11,12,13,14,‡,*] and Ryo Yamaguchi[1,15‡,*]

[1]Department of Advanced Transdisciplinary Sciences, Hokkaido University, Sapporo, Hokkaido, Japan. [2]interdisciplinary Biology Laboratory (iBLab), Division of Biological Science, Graduate School of Science, Nagoya University, Nagoya, Japan. [3]Department of Scientific Computing, Pukyong National University, Busan, South Korea. [4]Department of Mathematics, Pusan National University, Busan, South Korea. [5]Laboratory of RNA Viruses, Department of Virus Research, Institute for Life and Medical Sciences (LiMe), Kyoto University, Kyoto, Japan. [6]Mathematical Institute, University of Oxford, Oxford, United Kingdom. [7]Mathematics Institute, University of Warwick, Coventry, United Kingdom. [8]Zeeman Institute for Systems Biology and Infectious Disease Epidemiology Research, University of Warwick, Coventry, United Kingdom. [9]International Research Center for Neurointelligence, The University of Tokyo Institutes for Advanced Study, The University of Tokyo, Tokyo, Japan. [10]Institute of Mathematics for Industry, Kyushu University, Fukuoka, Japan. [11]Institute for the Advanced Study of Human Biology (ASHBi), Kyoto University, Kyoto, Japan. [12]Interdisciplinary Theoretical and Mathematical Sciences Program (iTHEMS), RIKEN, Saitama, Japan. [13]NEXT-Ganken Program, Japanese Foundation for Cancer Research (JFCR), Tokyo, Japan. [14]Science Groove Inc., Fukuoka, Japan. [15]Department of Zoology & Biodiversity Research Centre, University of British Columbia, Vancouver, British Columbia, Canada.

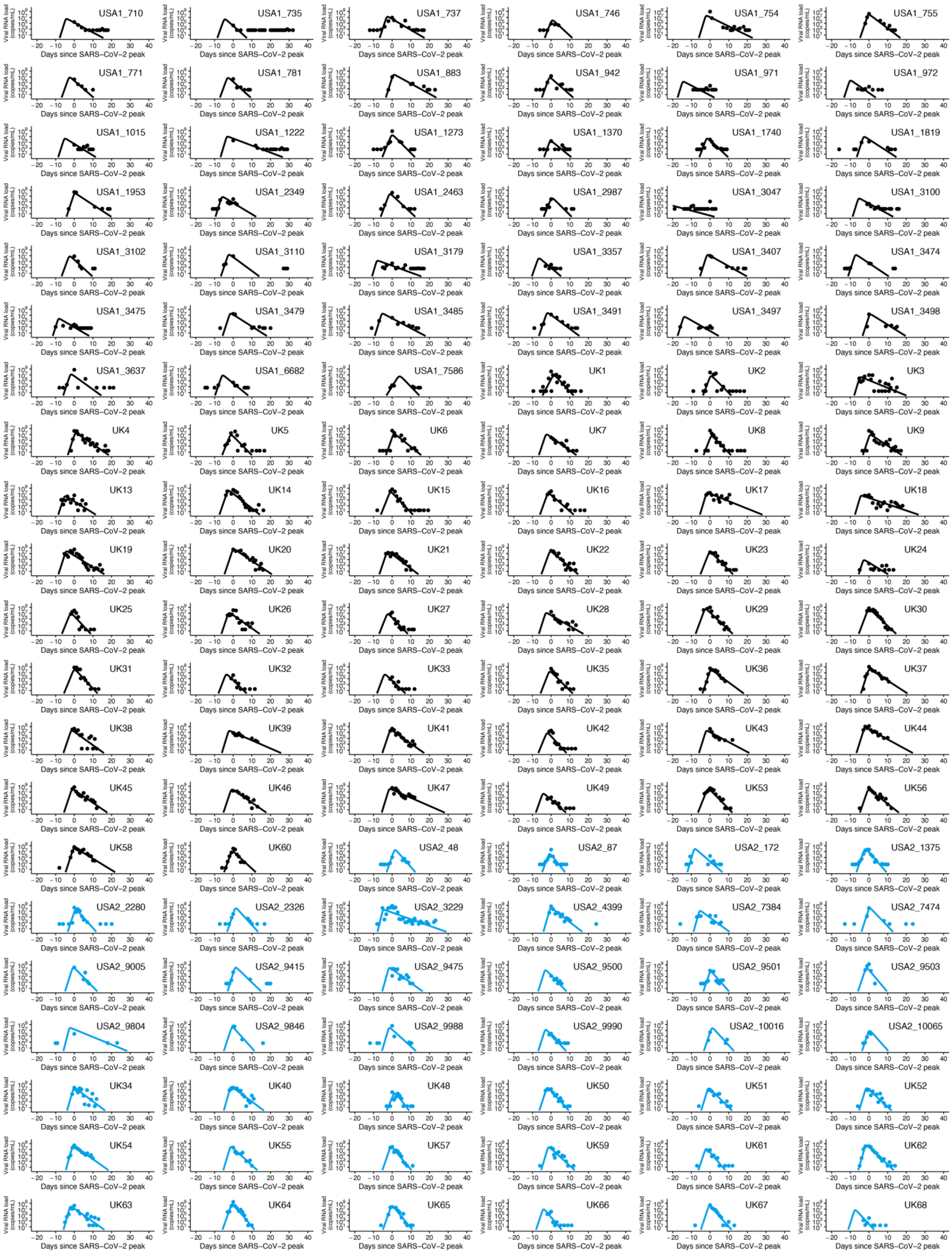
Viral RNA load (copies/mL)
Days since SARS−CoV−2 peak
USA1_710
USA1_735
USA1_737
USA1_746
USA1_754
USA1_755
USA1_771
USA1_781
USA1_883
USA1_942
USA1_971
USA1_972
USA1_1015
USA1_1222
USA1_1273
USA1_1370
USA1_1740
USA1_1819
USA1_1953
USA1_2349
USA1_2463
USA1_2987
USA1_3047
USA1_3100
USA1_3102
USA1_3110
USA1_3179
USA1_3357
USA1_3407
USA1_3474
USA1_3475
USA1_3479
USA1_3485
USA1_3491
USA1_3497
USA1_3498
USA1_3637
USA1_6682
USA1_7586
UK1
UK2
UK3
UK4
UK5
UK6
UK7
UK8
UK9
UK13
UK14
UK15
UK16
UK17
UK18
UK19
UK20
UK21
UK22
UK23
UK24
UK25
UK26
UK27
UK28
UK29
UK30
UK31
UK32
UK33
UK35
UK36
UK37
UK38
UK39
UK41
UK42
UK43
UK44
UK45
UK46
UK47
UK49
UK53
UK56
UK58
UK60
USA2_48
USA2_87
USA2_172
USA2_1375
USA2_2280
USA2_2326
USA2_3229
USA2_4399
USA2_7384
USA2_7474
USA2_9005
USA2_9415
USA2_9475
USA2_9500
USA2_9501
USA2_9503
USA2_9804
USA2_9846
USA2_9988
USA2_9990
USA2_10016
USA2_10065
UK34
UK40
UK48
UK50
UK51
UK52
UK54
UK55
UK57
UK59
UK61
UK62
UK63
UK64
UK65
UK66
UK67
UK68

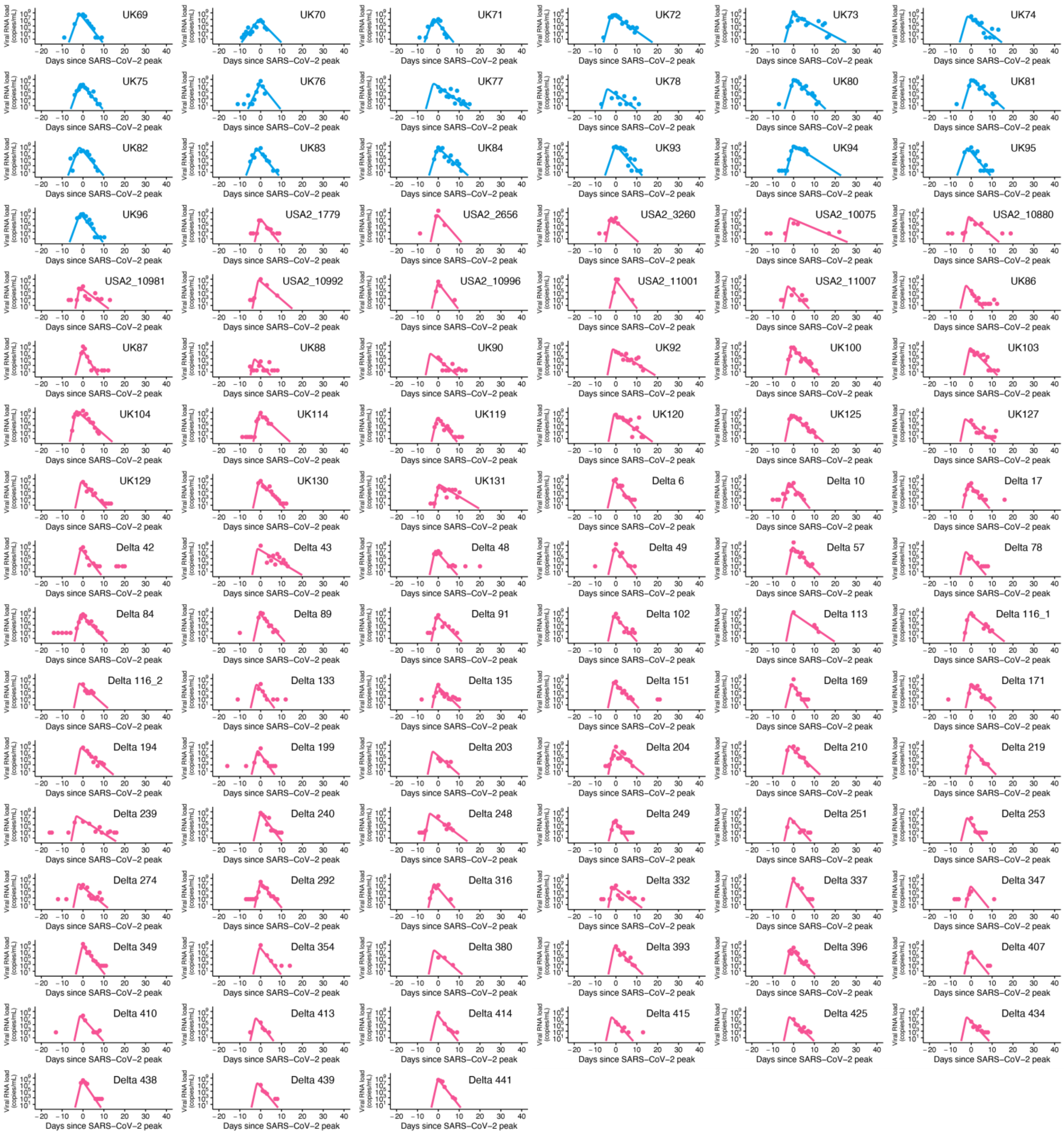

Figure S1. Viral load trajectory for individual patients infected with pre-Alpha, Alpha, and Delta variants. The estimated viral load for each individual patient (solid lines) along with the observed data (closed dots) are depicted using the best-fit parameter estimates. Pre-Alpha, Alpha, and Delta variants are shown in black, blue, and red, respectively. Source data are provided as a Source Data file.

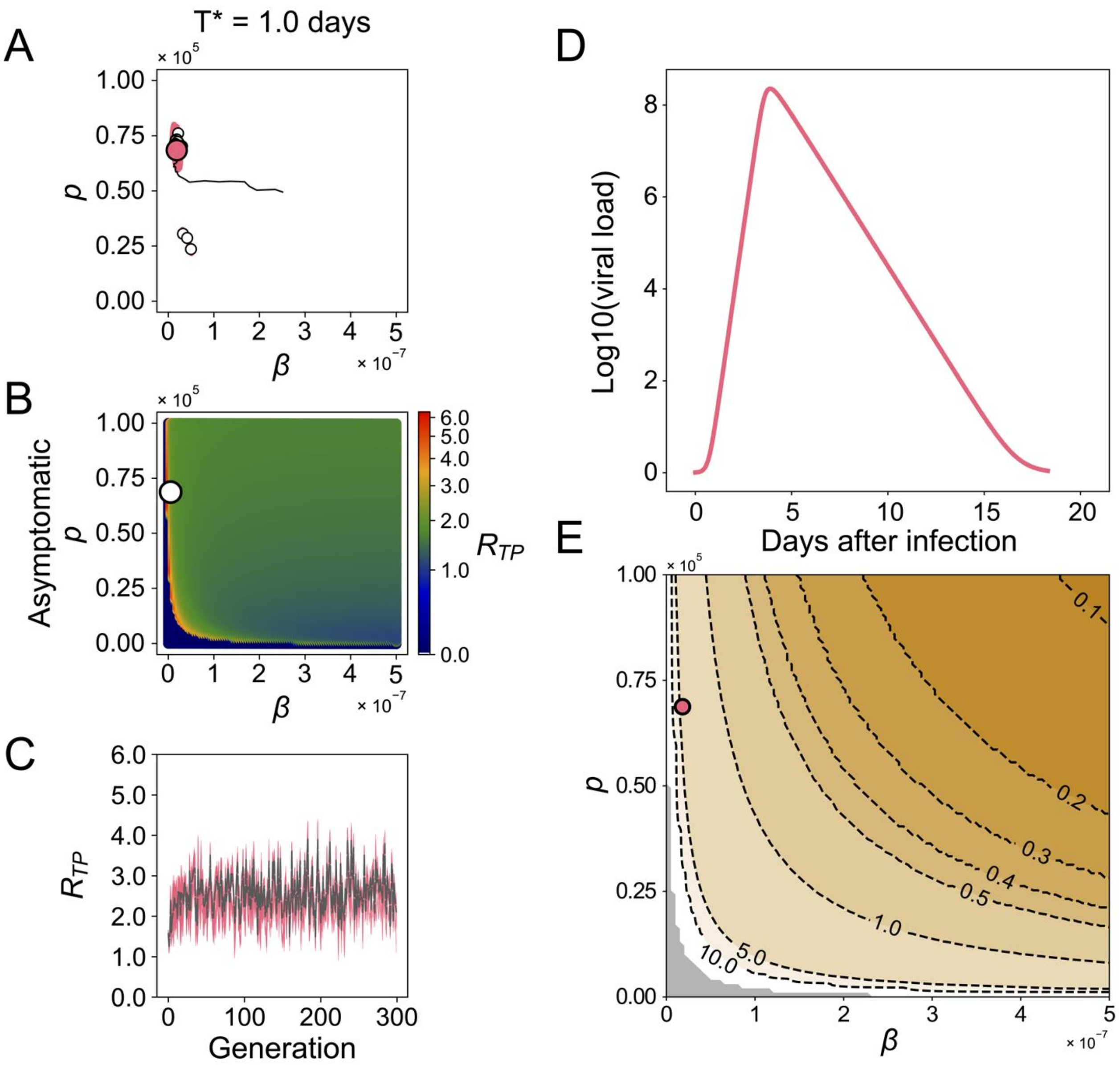

Figure S2. SARS-CoV-2 evolution *in silico* without symptomatic infection ($f = 0$ and $T^* = 1$): (A) Genetic algorithm (GA) exploring the evolutionary trajectories on the (β, p) plane until the generation of 300 is applied. Our choice of the incubation period, T^*, does not affect the calculation because no asymptomatic individuals change their transmissibility by isolation (i.e., no individual is applied to isolation). The white dots represent the endpoint of 100 independent simulation runs, and the contour lines are the kernel density estimation of their distribution. The colored dot in the panel is the mean value of the white dots, which represent the optimal set of (β, p) under the parameters we used. The black line is the mean trajectory of the GA through 300 generations. **(B)** The mean transmissibility fitness landscape aggregated from asymptomatic individuals (i.e., no symptomatic individuals due to $f = 0$) is described, using 100 runs of GA. The white dot represents the maximum

value of the mean transmissibility fitness, R_{TP}. **(C)** The trajectories of R_{TP} along the course of GA are calculated. The gray dotted lines are the mean trajectory over 100 trials of colored lines. **(D)** The time-series patterns of viral load with the optimal parameters of (β,p), which were obtained in (A), are shown. **(E)** The counter plot for the timing of peak viral load (i.e., peak time) is shown. The gray region is the parameter range satisfying $R_{TP} < 1$.

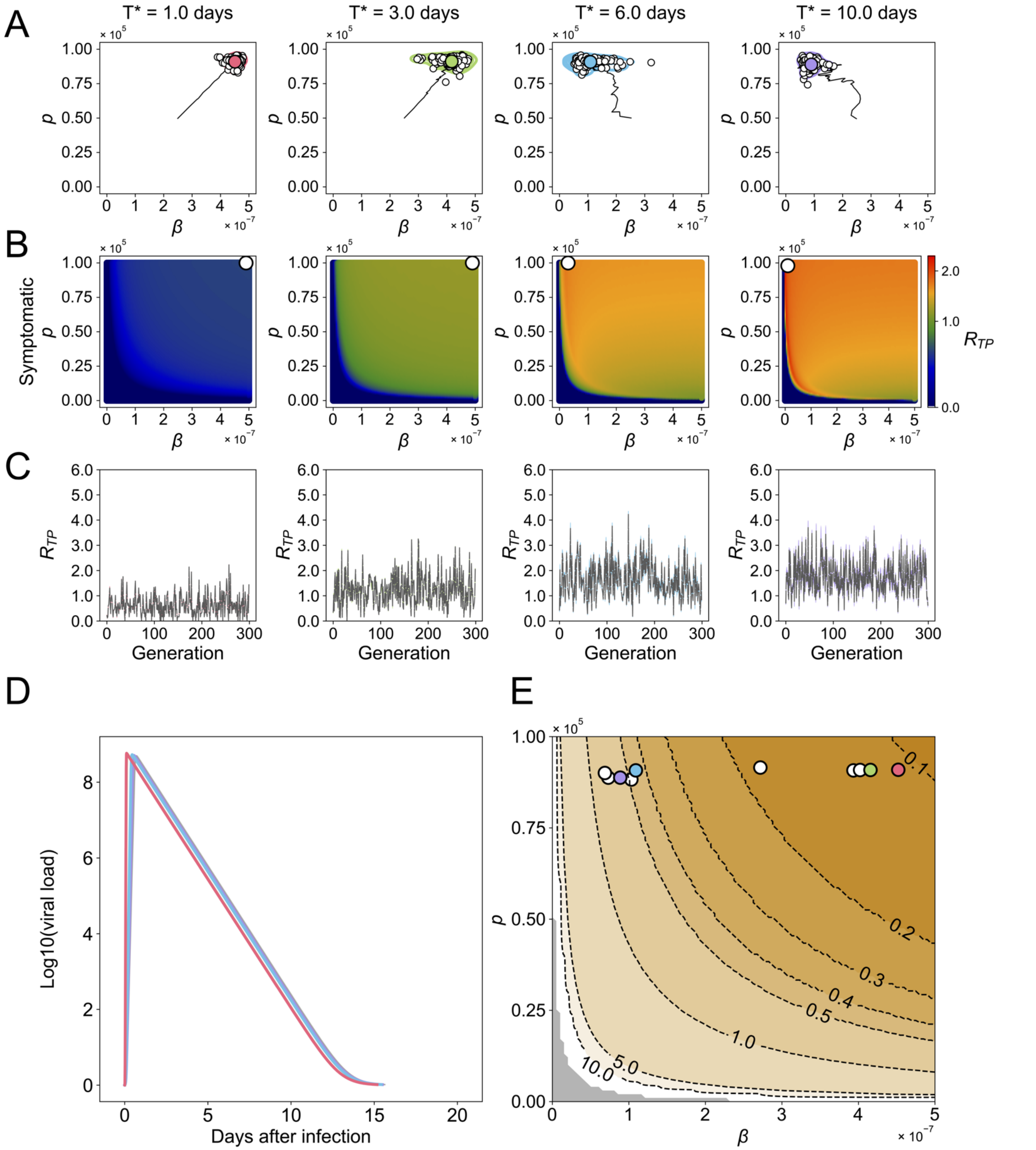

Figure S3. SARS-CoV-2 evolution *in silico* without asymptomatic infection ($f = 1$): (A) GA exploring the evolutionary trajectories on the (β, p) plane until the generation of 300 is applied, depending on different values of the incubation period, T^*. All individuals lose their transmissibility by isolation after the symptom onset ($T^* < t$). The white dots represent the endpoint of 100 independent

simulation runs, and the contour lines are the kernel density estimation of their distribution. The colored dot in each panel is the mean value of the white dots, which represents the optimal set of (β, p) under the parameters we used. The black line is the mean trajectory of the GA through 300 generations. **(B)** The mean transmissibility fitness landscapes aggregated from symptomatic individuals (i.e., no asymptomatic individuals due to $f = 0$) are described, using 100 runs of GA. The white dot represents the maximum value of the mean transmissibility fitness, R_{TP}. **(C)** The trajectories of R_{TP} along the course of GA with different T^* are calculated. The gray dotted lines are the mean trajectory over 100 trials of colored lines. **(D)** The time-series patterns of viral load with the optimal parameters of (β, p) with different T^*, which were obtained in (A), are shown. Each curve is colored accordingly. **(E)** The counter plot for the timing of peak viral load is shown. Each dot is colored accordingly. The gray region is the parameter range satisfying $R_{TP} < 1$.

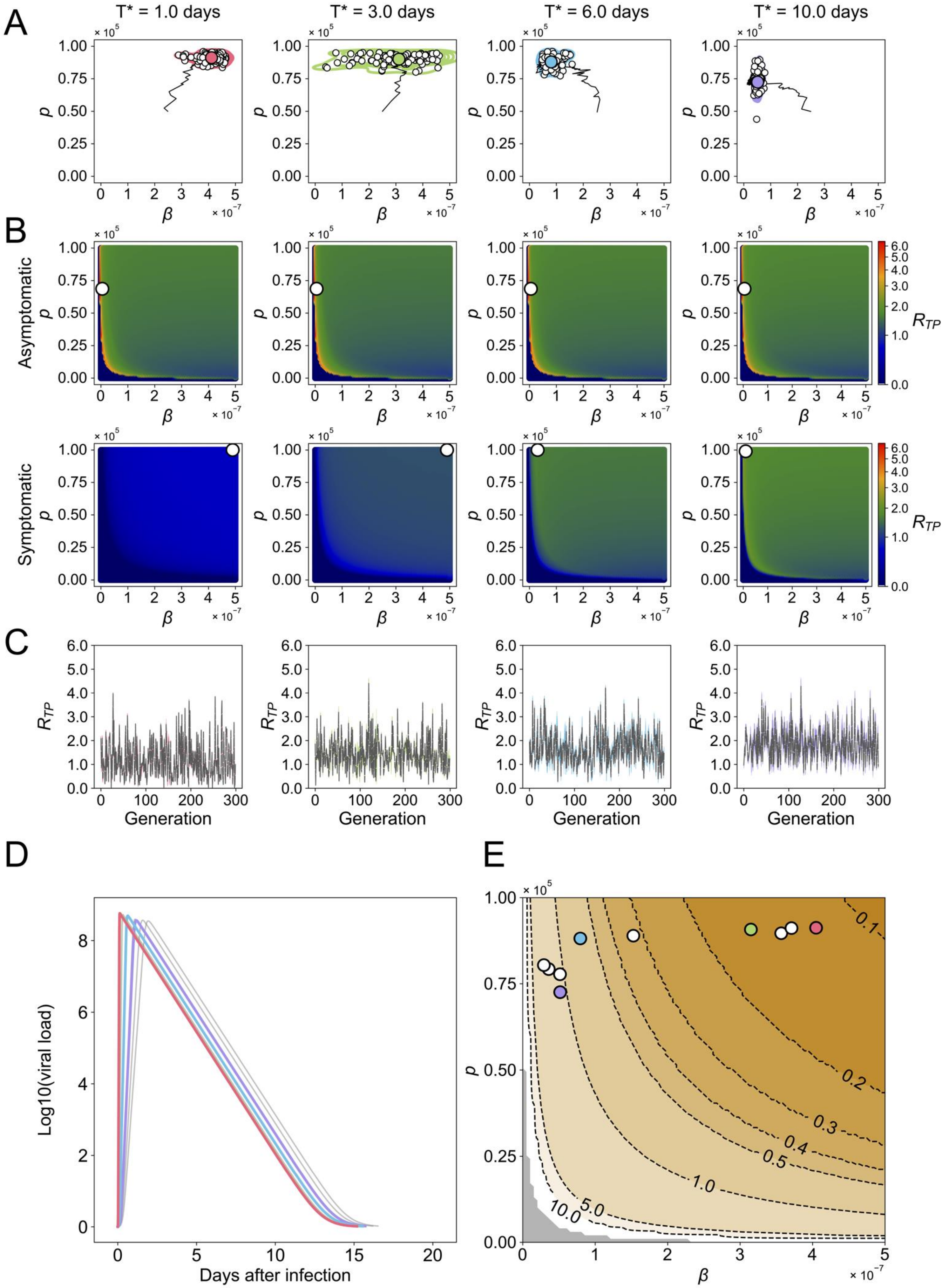
A
B
C
D
E
T* = 1.0 days
T* = 3.0 days
T* = 6.0 days
T* = 10.0 days
Asymptomatic
Symptomatic
R_{TP}
Generation
Log10(viral load)
Days after infection
0.1
0.2
0.3
0.4
0.5
1.0
5.0
10.0

Figure S4-1. Sensitivity analysis of the proportion of symptomatic infection ($\boldsymbol{f = 0.5}$): (A) Genetic algorithm (GA) exploring the evolutionary trajectories on the (β,p) plane until the generation of 300 is applied, depending on different values of the incubation period, T^*. All individuals lose their transmissibility by NPIs after symptom onset ($T^* < t$). The white dots represent the endpoint of 100 independent simulation runs, and the contour lines are the kernel density estimation of their distribution. The colored dot in each panel is the mean value of the white dots, which represent the optimal set of (β,p) under the parameters we used. The black line is the mean trajectory of the GA through 300 generations. **(B)** The mean transmissibility fitness landscape aggregated solely from the asymptomatic (top row) and symptomatic (bottom row) individuals, respectively, are described, using 100 runs of GA. The white dot represents the maximum value of the mean transmissibility fitness, R_{TP}. **(C)** The trajectories of R_{TP} along the course of GA with different T^* are calculated. The gray dotted lines are the mean trajectory over 100 trials of colored lines. **(D)** The time-series patterns of viral load with the optimal parameters of (β,p) with different T^*, which were obtained in (A), are shown. Each curve is colored accordingly. **(E)** The counter plot for the timing of peak viral load is shown. Each dot is colored accordingly. The gray region is the parameter range satisfying $R_{TP} < 1$.

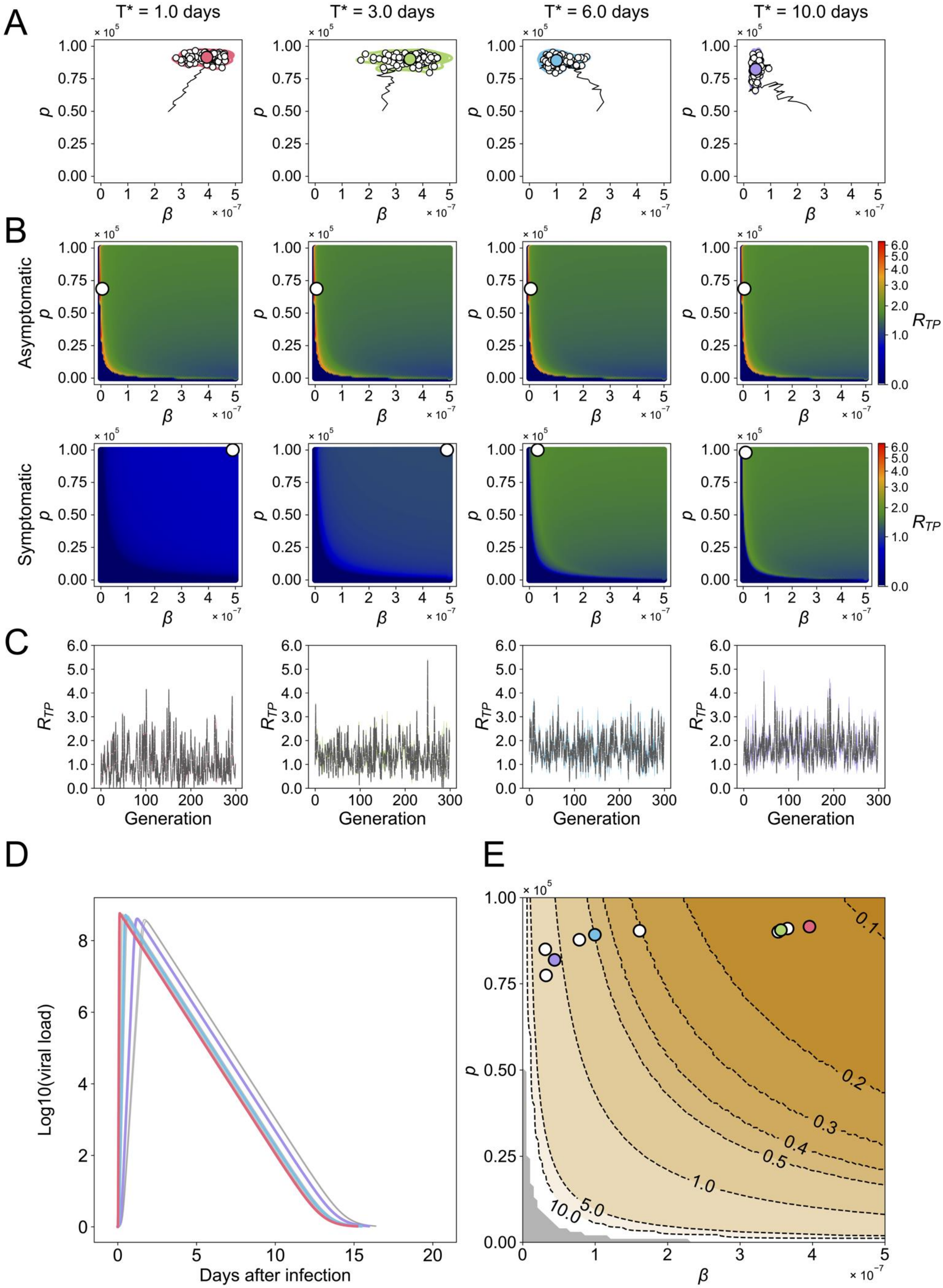
A
T* = 1.0 days
T* = 3.0 days
T* = 6.0 days
T* = 10.0 days
p
β
× 10^5
× 10^-7
B
Asymptomatic
Symptomatic
R_TP
C
R_TP
Generation
D
Log10(viral load)
Days after infection
E
p
β
10.0
5.0
1.0
0.5
0.4
0.3
0.2
0.1

Figure S4-2. Sensitivity analysis of the proportion of symptomatic infection ($f = 0.6$): Same explanation for **(A-E)** is applied as in **Figure S4-1**.

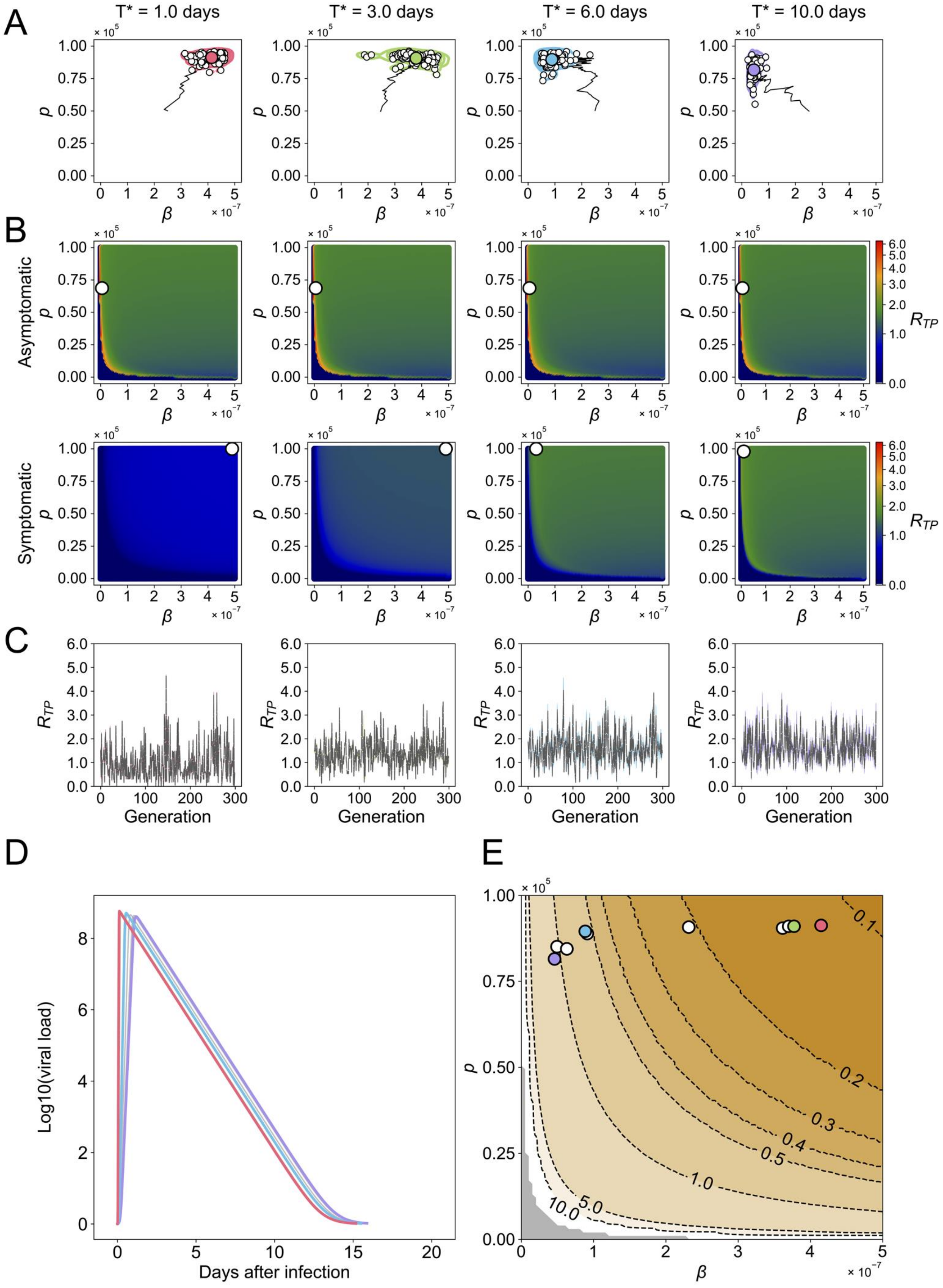
A
T* = 1.0 days
T* = 3.0 days
T* = 6.0 days
T* = 10.0 days
B
Asymptomatic
Symptomatic
R_{TP}
C
Generation
D
Log10(viral load)
Days after infection
E
p
β
× 10^5
× 10^-7
0.1
0.2
0.3
0.4
0.5
1.0
5.0
10.0

Figure S4-3. Sensitivity analysis of the proportion of symptomatic infection ($f = 0.7$): Same explanation for **(A-E)** is applied as in **Figure S4-1**.

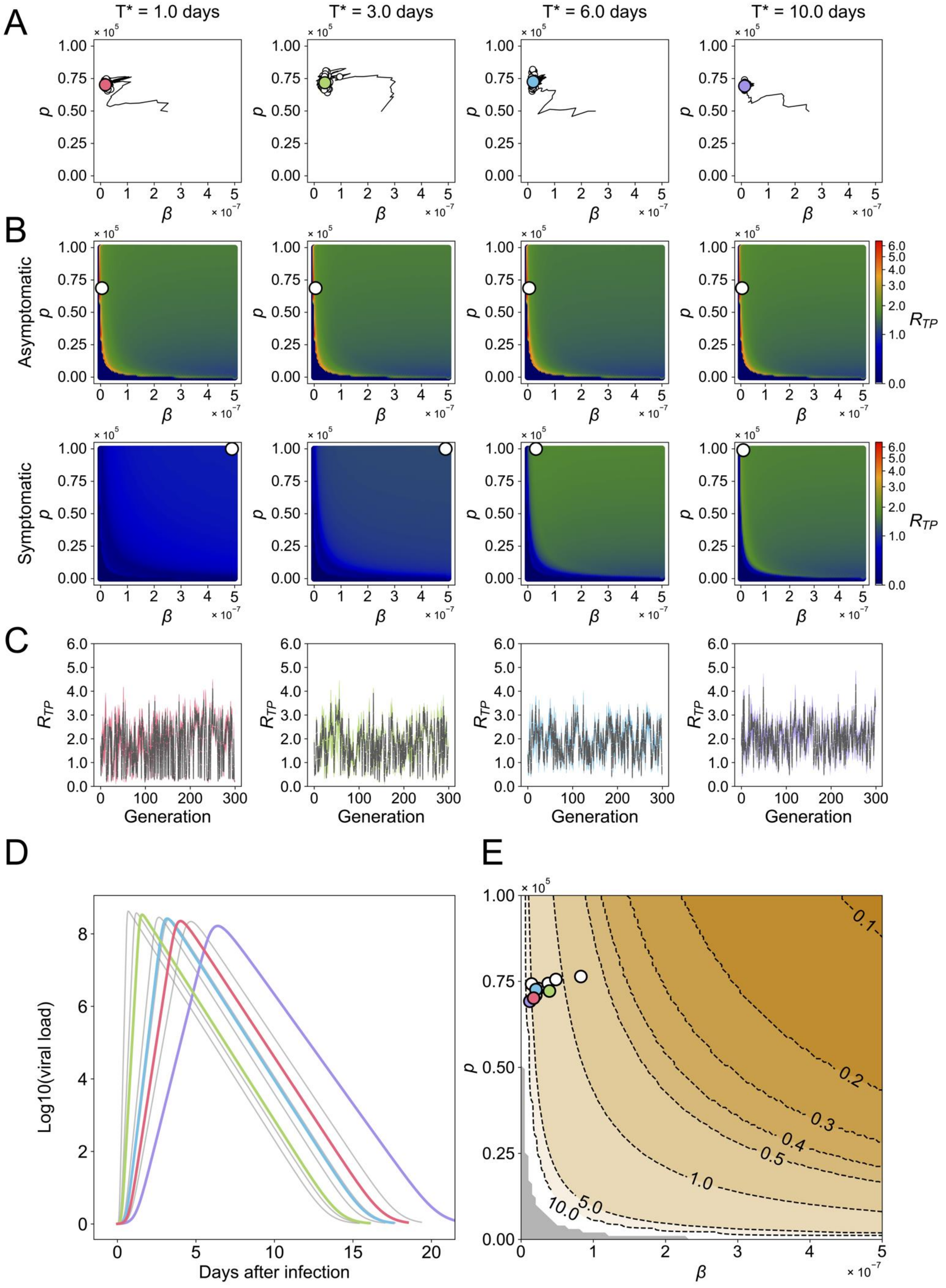
A
B
C
D
E
T* = 1.0 days
T* = 3.0 days
T* = 6.0 days
T* = 10.0 days
× 10^5
× 10^-7
p
β
Asymptomatic
Symptomatic
R_{TP}
Generation
Log10(viral load)
Days after infection
0.1
0.2
0.3
0.4
0.5
1.0
5.0
10.0

Figure S5-1. Sensitivity analysis of the impact of Incomplete Isolation on Viral Load Dynamics ($\boldsymbol{f = 0.3}$ and $\boldsymbol{\xi = 0.1}$): (A) Genetic algorithm (GA) exploring the evolutionary trajectories on the (β, p) plane until the generation of 300 is applied, depending on different values of the incubation period, T^*. All individuals lose their transmissibility by NPIs after symptom onset ($T^* < t$). The white dots represent the endpoint of 100 independent simulation runs, and the contour lines are the kernel density estimation of their distribution. The colored dot in each panel is the mean value of the white dots, which represent the optimal set of (β, p) under the parameters we used. The black line is the mean trajectory of the GA through 300 generations. **(B)** The mean transmissibility fitness landscape aggregated solely from the asymptomatic (top row) and symptomatic (bottom row) individuals, respectively, are described, using 100 runs of GA. The white dot represents the maximum value of the mean transmissibility fitness, R_{TP}. **(C)** The trajectories of R_{TP} along the course of GA with different T^* are calculated. The gray dotted lines are the mean trajectory over 100 trials of colored lines. **(D)** The time-series patterns of viral load with the optimal parameters of (β, p) with different T^*, which were obtained in (A), are shown. Each curve is colored accordingly. **(E)** The counter plot for the timing of peak viral load is shown. Each dot is colored accordingly. The gray region is the parameter range satisfying $R_{TP} < 1$. Additional graphs illustrating dependency on parameter f: see https://zenodo.org/record/10030531

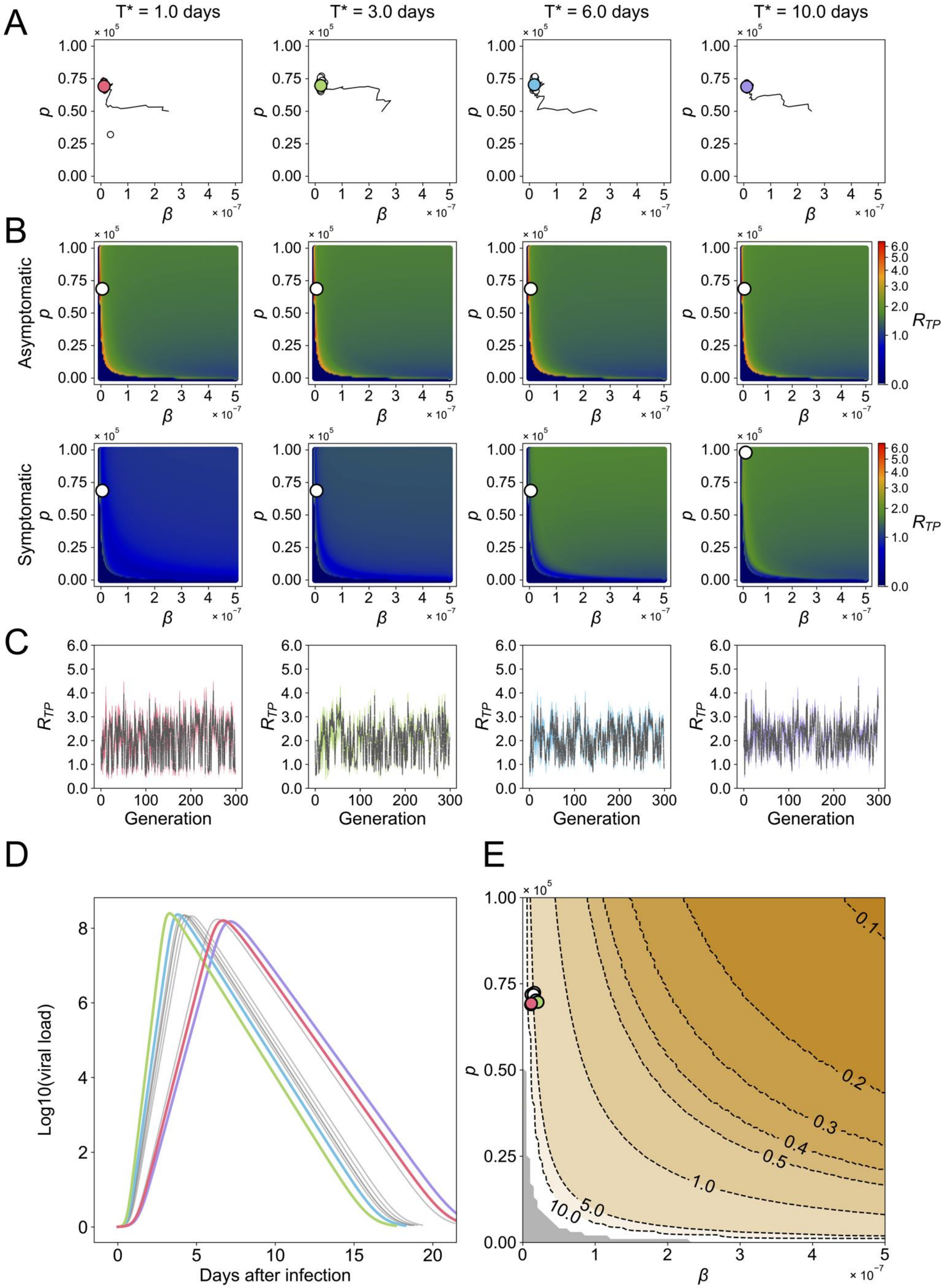
A
B
C
D
E
T* = 1.0 days
T* = 3.0 days
T* = 6.0 days
T* = 10.0 days
Asymptomatic
Symptomatic
p
β
R_{TP}
Generation
Log10(viral load)
Days after infection
0.1
0.2
0.3
0.4
0.5
1.0
5.0
10.0

Figure S5-2. Sensitivity analysis of the impact of Incomplete Isolation on Viral Load Dynamics ($\boldsymbol{f = 0.3}$ and $\boldsymbol{\xi = 0.3}$): Same explanation for **(A-E)** is applied as in **Figure S5-1**.

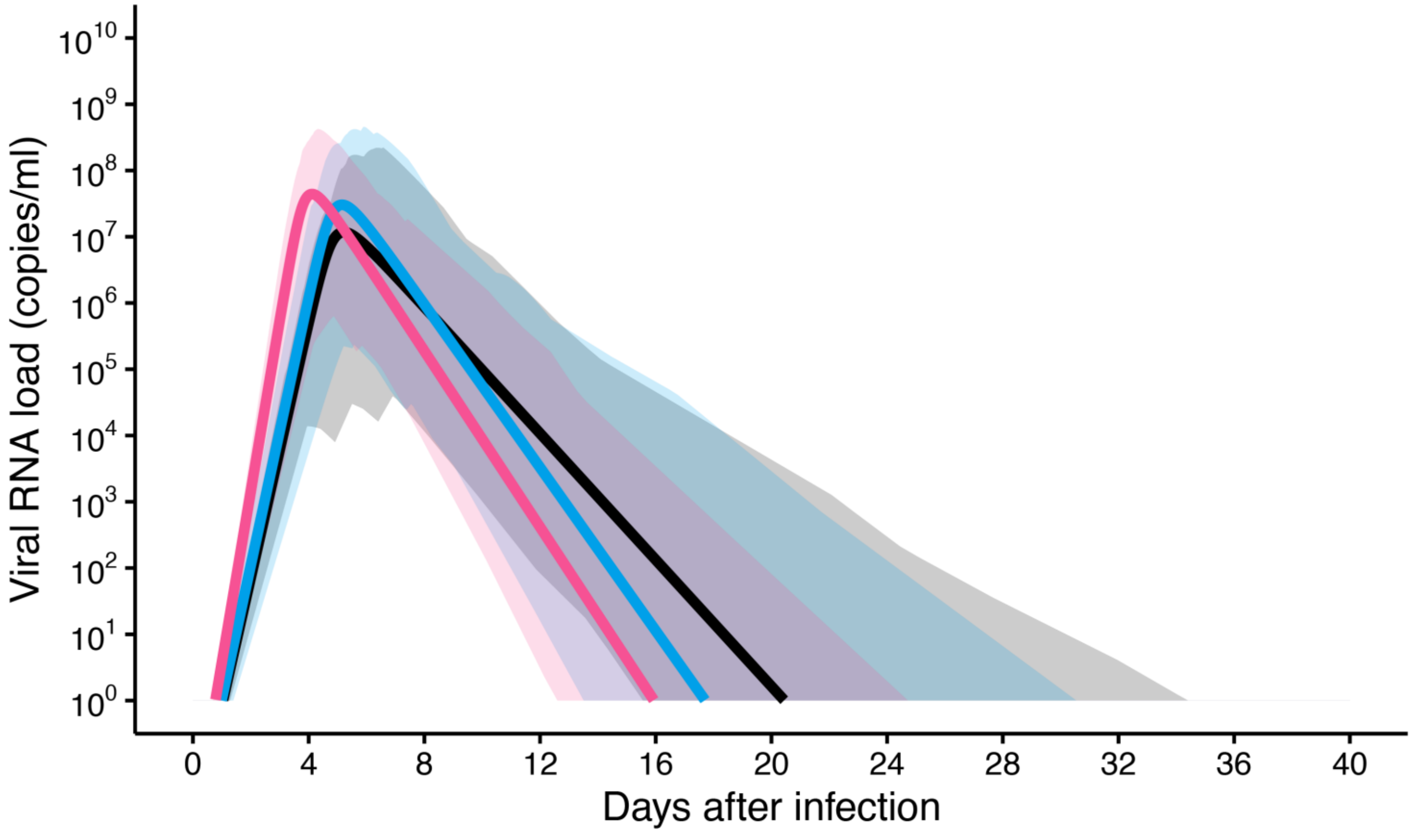

Figure S6. Quantification of SARS-CoV-2 infection dynamics with patients who have prior immunity: The inferred viral dynamics of 293 COVID-19 patients with and without prior immunity. It contains 225 patients without prior immunity (86 with pre-Alpha variant, 59 with Alpha variant and 80 with Delta variant), and 68 patients with prior immunity by vaccination or pre-infection (14 with Alpha variant and 54 with Delta variant). The solid curves correspond to the solution of Eqs.(4-5) using the best-fit population parameters, and the shadow regions are the 95% interquantile range of the predictive interval using the estimated parameters for each patient. The black, blue, and red colors indicate the pre-Alpha, Alpha, and Delta variants, respectively.

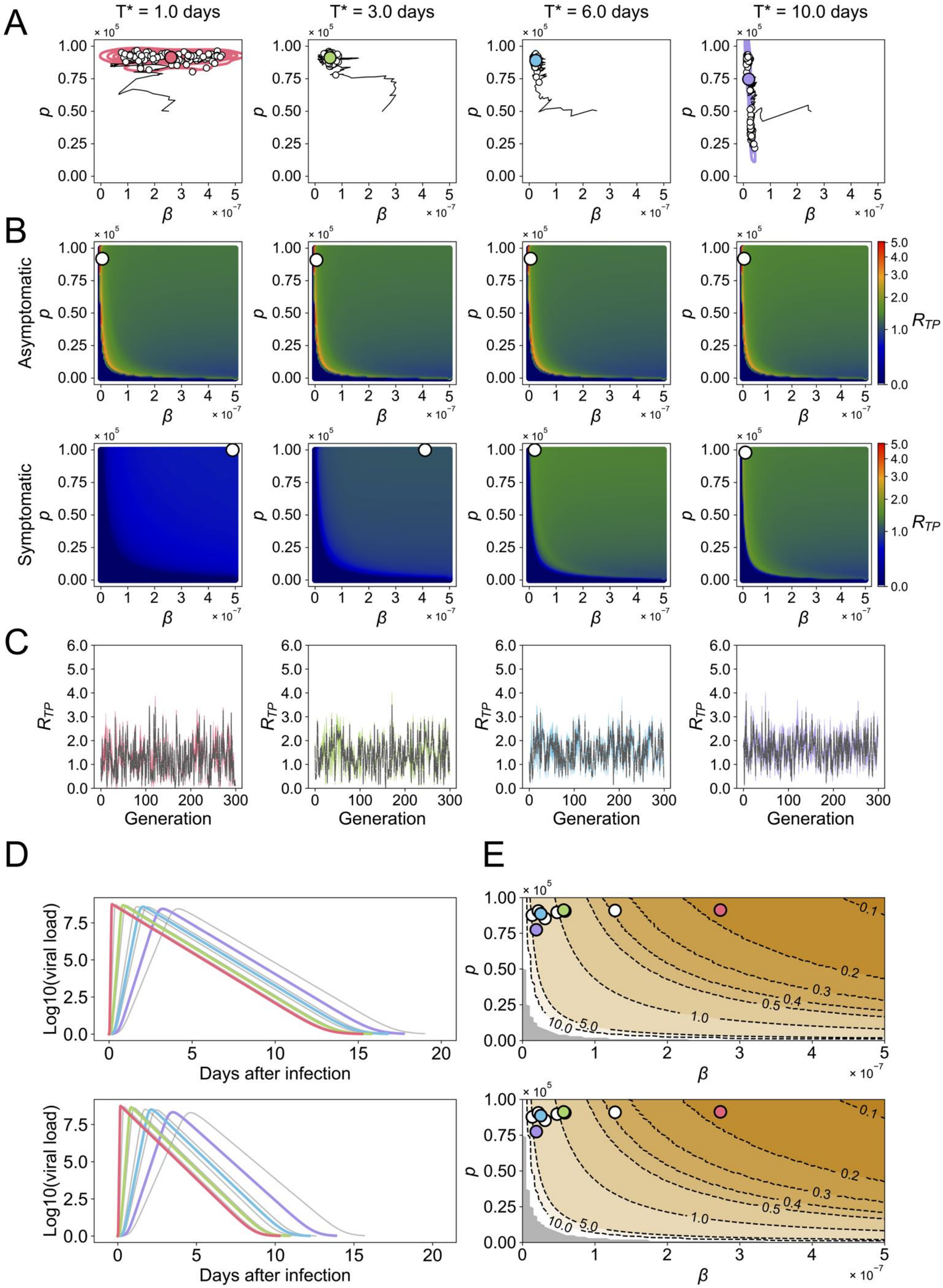

A
B
C
D
E
T* = 1.0 days
T* = 3.0 days
T* = 6.0 days
T* = 10.0 days
Asymptomatic
Symptomatic
R_{TP}
Generation
Log10(viral load)
Days after infection
10.0
5.0
1.0
0.5
0.4
0.3
0.2
0.1

Figure S7-1. Sensitivity analysis of effects of vaccination rates on optimal viral parameters ($f = 0.3$ and vaccination rate $= 0.5$): (A) Genetic algorithm (GA) exploring the evolutionary trajectories on the (β,p) plane until the generation of 300 is applied, depending on different values of the incubation period, T^*. All individuals lose their transmissibility by NPIs after symptom onset ($T^* < t$). The white dots represent the endpoint of 100 independent simulation runs, and the contour lines are the kernel density estimation of their distribution. The colored dot in each panel is the mean value of the white dots, which represent the optimal set of (β,p) under the parameters we used. The black line is the mean trajectory of the GA through 300 generations. **(B)** The mean transmissibility fitness landscape aggregated solely from the asymptomatic (top row) and symptomatic (bottom row) individuals, respectively, are described, using 100 runs of GA. The white dot represents the maximum value of the mean transmissibility fitness, R_{TP}. **(C)** The trajectories of R_{TP} along the course of GA with different T^* are calculated. The gray dotted lines are the mean trajectory over 100 trials of colored lines. **(D)** The time-series patterns of viral load with the optimal parameters of (β,p) with different T^*, which were obtained in (A), are shown. Each curve is colored accordingly. The upper row represents the case for unvaccinated individuals, while the lower row demonstrates the case for vaccinated individuals (with δ increased by 1.5 times). **(E)** The counter plot for the timing of peak viral load is shown. Each dot is colored accordingly. The gray region is the parameter range satisfying $R_{TP} < 1$. The upper and lower rows are the same as in (D). Additional graphs illustrating dependency on parameter f: see https://zenodo.org/record/10030531

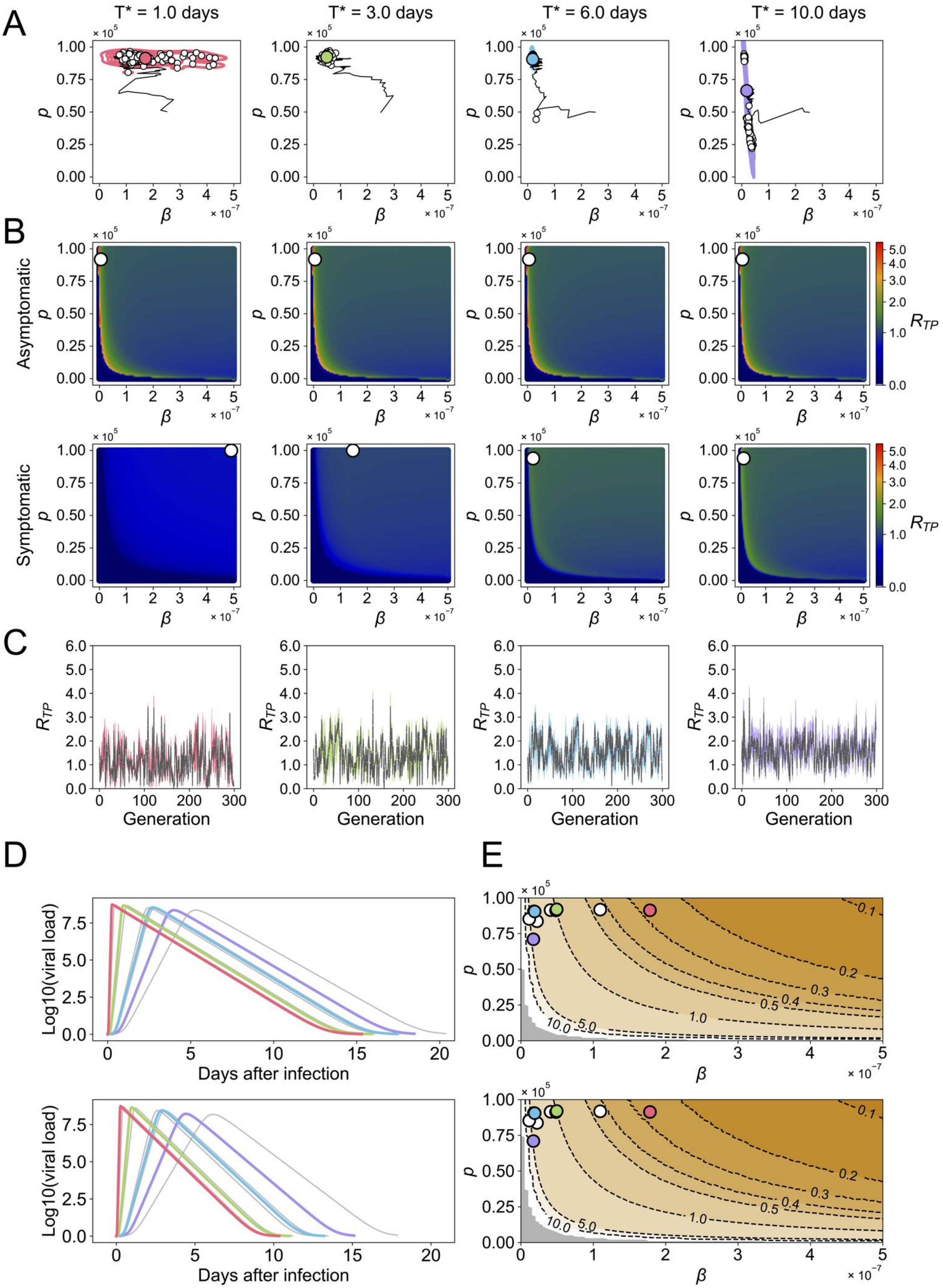
A
B
C
D
E
T* = 1.0 days
T* = 3.0 days
T* = 6.0 days
T* = 10.0 days
Asymptomatic
Symptomatic
p
β
R_TP
Generation
Log10(viral load)
Days after infection

Figure S7-2. Sensitivity analysis of effects of vaccination rates on optimal viral parameters ($f = 0.3$ and vaccination rate $= 0.8$): Same explanation for **(A-E)** is applied as in **Figure S6-1**.

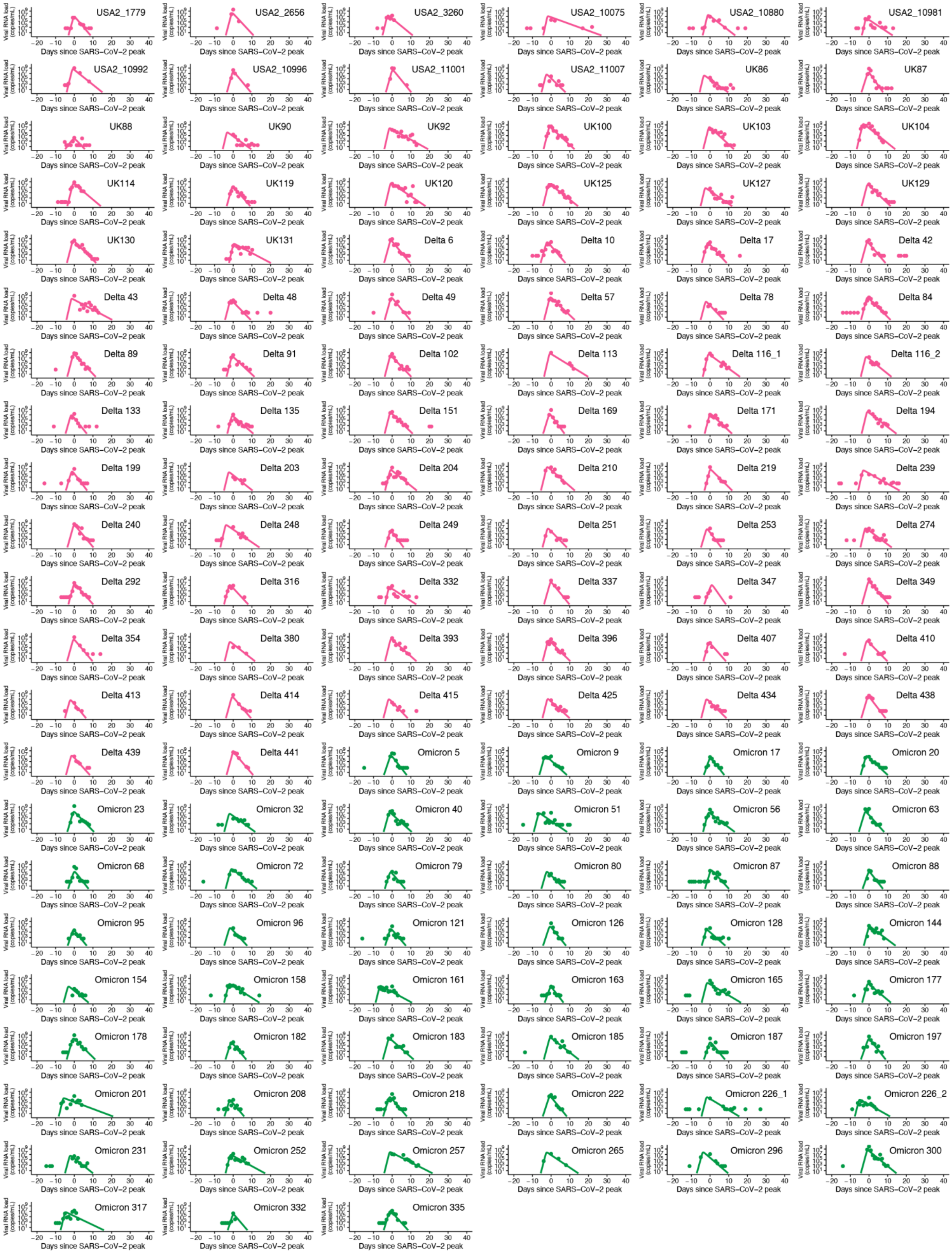

USA2_1779
USA2_2656
USA2_3260
USA2_10075
USA2_10880
USA2_10981
USA2_10992
USA2_10996
USA2_11001
USA2_11007
UK86
UK87
UK88
UK90
UK92
UK100
UK103
UK104
UK114
UK119
UK120
UK125
UK127
UK129
UK130
UK131
Delta 6
Delta 10
Delta 17
Delta 42
Delta 43
Delta 48
Delta 49
Delta 57
Delta 78
Delta 84
Delta 89
Delta 91
Delta 102
Delta 113
Delta 116_1
Delta 116_2
Delta 133
Delta 135
Delta 151
Delta 169
Delta 171
Delta 194
Delta 199
Delta 203
Delta 204
Delta 210
Delta 219
Delta 239
Delta 240
Delta 248
Delta 249
Delta 251
Delta 253
Delta 274
Delta 292
Delta 316
Delta 332
Delta 337
Delta 347
Delta 349
Delta 354
Delta 380
Delta 393
Delta 396
Delta 407
Delta 410
Delta 413
Delta 414
Delta 415
Delta 425
Delta 434
Delta 438
Delta 439
Delta 441
Omicron 5
Omicron 9
Omicron 17
Omicron 20
Omicron 23
Omicron 32
Omicron 40
Omicron 51
Omicron 56
Omicron 63
Omicron 68
Omicron 72
Omicron 79
Omicron 80
Omicron 87
Omicron 88
Omicron 95
Omicron 96
Omicron 121
Omicron 126
Omicron 128
Omicron 144
Omicron 154
Omicron 158
Omicron 161
Omicron 163
Omicron 165
Omicron 177
Omicron 178
Omicron 182
Omicron 183
Omicron 185
Omicron 187
Omicron 197
Omicron 201
Omicron 208
Omicron 218
Omicron 222
Omicron 226_1
Omicron 226_2
Omicron 231
Omicron 252
Omicron 257
Omicron 265
Omicron 296
Omicron 300
Omicron 317
Omicron 332
Omicron 335
Viral RNA load (copies/mL)
Days since SARS−CoV−2 peak

Figure S8. Viral load trajectory for individual patients infected with Delta and Omicron variants: The estimated viral load for each individual patient (solid lines) along with the observed data (closed dots) are depicted using the best-fit parameter estimates. Delta and Omicron variants are shown in red and green, respectively. The data for the delta variant are same as in **Figure S1**. Source data are provided as a Source Data file.

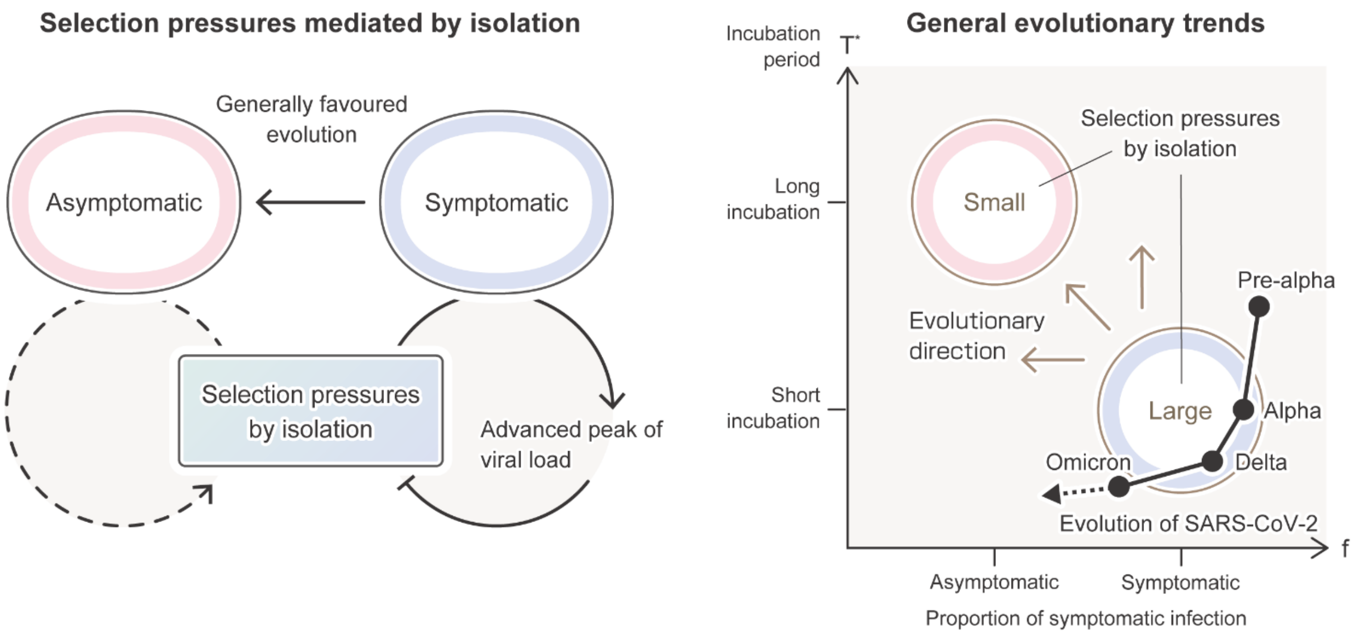

Figure S9. Possible mechanisms of adaptive virus response under isolation: Selection pressures mediated by isolation (left panel) and general evolutionary trends (right panel) are described. The solid circular arrow in the left panel represents that isolation can be a substantial selective force only when a focal patient is symptomatic. In this case, the advanced viral peak evolves to mitigate the selection pressure. However, asymptomatic infection is always favored from a perspective of virus evolution that maximizes the number of secondary infectious individuals because isolation does not limit transmissions. Thus, the dashed circular arrow in the left panel shows that no selective force is generated by isolation when the focal patient is asymptomatic. In the right panel, the possible general evolutionary trends show that a more extended incubation period (i.e., larger T^*) and/or a higher proportion of asymptomatic infection (i.e., smaller f) may generally evolve to mitigate the selection pressure of isolation. The black trajectory represents evolutionary transitions among the pre-Alpha, Alpha, Delta, and Omicron variants of SARS-CoV-2 in our analysis. The incubation period decreases as SARS-CoV-2 mutates, implying the selection pressure by isolation, and therefore the peak viral load is advanced as observed in the Delta variant. In addition, a high proportion of asymptomatic infections has recently evolved. It is also noteworthy that once the higher proportion of asymptomatic infection evolves, other viral phenotypic evolution, such as slower and/or lower peak viral load, may be allowed to evolve as observed in the Omicron variant. These multiple evolutionary outcomes may be counterbalanced during viral evolution under human behavior change (see the main text for further details).

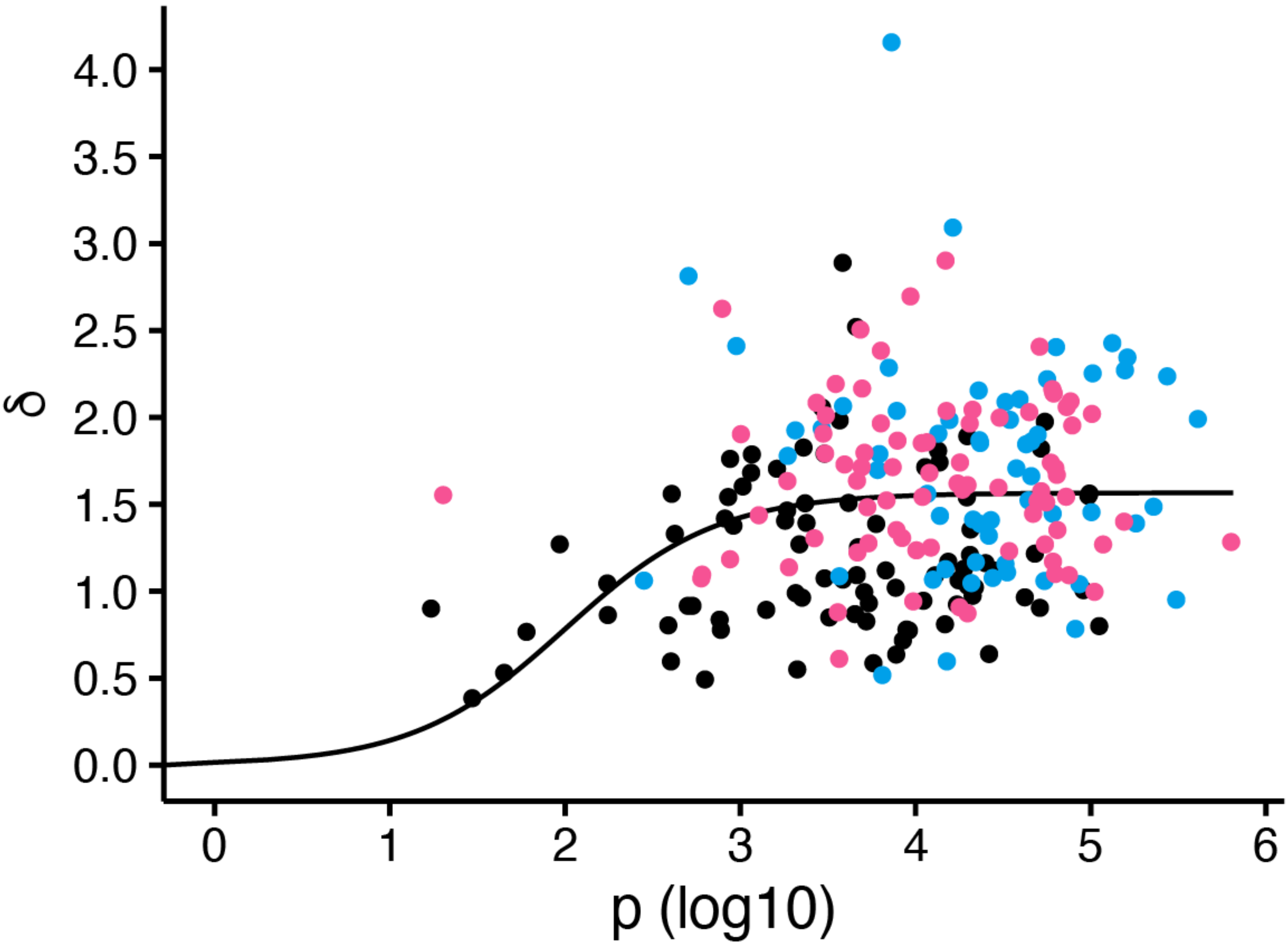

Figure S10. Relation between viral production and death rate of infected cells. Each dot shows the estimated values of p and δ for each individual for infection with the pre-Alpha, Alpha, and Delta strains. The solid line is the best-fitting curve for $\delta = \delta_{\max} p/(p + p_{50})$, whose root mean square error (RMSE) is 0.4. Pre-Alpha, Alpha, and Delta variants are colored in black, blue, and red, respectively. Source data are provided as a Source Data file.

Table S1. Summary of the study data

Country	Number of cases	Specimens	Detection limit	Reference
**Pre-Alpha**				
USA-1	39	Upper respiratory tract	2.66 log10 (copies/ml)	(1)
UK	47	Upper respiratory tract	1.49 log10 (copies/ml)	(2)
**Alpha**				
USA-2	22	Upper respiratory tract	2.66 log10 (copies/ml)	(3)
UK	37	Upper respiratory tract	1.49 log10 (copies/ml)	(2)
**Delta**				
USA-2	10	Upper respiratory tract	2.66 log10 (copies/ml)	(3)
USA-3	54	Upper respiratory tract	2.66 log10 (copies/ml)	(4)
UK	16	Upper respiratory tract	1.49 log10 (copies/ml)	(2)
**Omicron**				
USA-3	49	Upper respiratory tract	2.66 log10 (copies/ml)	(4)

Table S2. Estimated fixed parameters for SARS-CoV-2 pre-Alpha, Alpha, and Delta variants

Parameters	Symbol	Unit	Pre-Alpha	Alpha	Delta
Maximum rate constant for viral replication	γ	day^{-1}	5.43	$6.31^{\#}$	$8.51^{\#}$
Rate constant for virus infection	β	(copies/mL)$^{-1}$ day^{-1}	2.33×10^{-7}	$4.74 \times 10^{-8\#}$	1.03×10^{-7}
Death rate of infected cells	δ	day^{-1}	1.11	$1.57^{\#}$	$1.53^{\#}$
Time from infection to peak viral load	T_p	days	7.49	$6.49^{\#}$	$4.81^{\#}$

[#] Statistically different from pre-Alpha variant (the Wald test).

Table S3. Fixed parameters for multi-level population dynamics model

Parameters	Symbol	Value	Reference
The number of uninfected target cells at initial time	$T(0)$	133000	(5)
The clearance rate of the virus	c	20	(6)
The fraction of uninfected target cells compared with that at initial time	$f(0)$	1	(6)
The amount of the virus at initial time	$V(0)$	0.01	(5)
The maximum value of δ	δ_{max}	1.57	Estimated from data
The viral production rate satisfying $\delta = \delta_{\max}/2$	p_{50}	100	Estimated from data
The maximum duration of viral shedding	D_{max}	80	Estimated from data
The total viral load at which the duration is half of its maximum	V_{50}	198.58	Estimated from data
The steepness at which duration increases with increasing viral load	D_k	1.17	Estimated from data
The shape parameter of gamma distribution	k	1.5	Fixed as an example
The scale parameter of gamma distribution	θ	1.5	Fixed as an example
The maximum value of β	β_{max}	5.0×10^{-7}	Fixed as an example
The maximum value of p	p_{max}	1.0×10^{5}	Fixed as an example

Table S4. Fixed parameters for genetic algorithm (GA)

**Parameters**	**Value**
The population size	100
The number of iterations	300
The number of elitism	2
The probability of selection	1.0
The probability of crossover	0.2
The probability of mutation	0.7
The predefined range of β in mutation	0.5×10^{-7}
The predefined range of p in mutation	0.1×10^{5}

Table S5. Estimated fixed parameters for SARS-CoV-2 Delta and Omicron variants

Parameters	Symbol	Unit	Delta	Omicron
Maximum rate constant for viral replication	γ	day^{-1}	7.83	$7.21^{\#}$
Rate constant for virus infection	β	$(copies/mL)^{-1}$ day^{-1}	1.23×10^{-7}	$5.38 \times 10^{-7\#}$
Death rate of infected cells	δ	day^{-1}	1.60	1.49
Time from infection to peak viral load	T_p	days	4.96	5.46

$^{\#}$ Statistically different from delta variant (the Wald test).

Algorithm S1. Procedure of genetic algorithm (GA)

**Genetic algorithm**
Input: s_i, solution i of combinations of β and p
Input: P(t = i) = {s_1, s_2, …, s_j}, population of solutions in iteration i
t ← 0;
Initialize (P(t = 0)) with random β and p;
Evaluate (P(t = 0));
while not termination do
The scale parameter of gamma distribution
$P(t)_e$ ← Elitism (P(t));
$P(t)_p$ ← selectSolutions (P(t));
$P(t)_c$ ← crossover ($P(t)_e$ +$P(t)_p$);
Mutate ($P(t)_c$);
Evaluate ($P(t)_c$);
P(t+1) ← $P(t)_c$;
t ← t + 1;
end while

References

1. S. M. Kissler *et al.*, Viral dynamics of acute SARS-CoV-2 infection and applications to diagnostic and public health strategies. *PLoS Biol* **19**, e3001333 (2021).
2. A. Singanayagam *et al.*, Community transmission and viral load kinetics of the SARS-CoV-2 delta (B.1.617.2) variant in vaccinated and unvaccinated individuals in the UK: a prospective, longitudinal, cohort study. *Lancet Infect Dis* **22**, 183-195 (2022).
3. S. M. Kissler *et al.*, Viral Dynamics of SARS-CoV-2 Variants in Vaccinated and Unvaccinated Persons. *N Engl J Med* **385**, 2489-2491 (2021).
4. J. A. Hay *et al.*, Viral dynamics and duration of PCR positivity of the SARS-CoV-2 Omicron variant. *medRxiv* 10.1101/2022.01.13.22269257, 2022.2001.2013.22269257 (2022).
5. A. Gonçalves *et al.*, Timing of Antiviral Treatment Initiation is Critical to Reduce SARS-CoV-2 Viral Load. *CPT Pharmacometrics Syst Pharmacol* **9**, 509-514 (2020).
6. S. Iwanami *et al.*, Detection of significant antiviral drug effects on COVID-19 with reasonable sample sizes in randomized controlled trials: A modeling study. *PLoS Med* **18**, e1003660 (2021).
